# Infection Highlights 2000-01

**DOI:** 10.3201/eid0804.020044

**Published:** 2002-04

**Authors:** James P. Steinberg

**Affiliations:** Emory University School of Medicine

This 98-page paperback in the Fast Facts series contains concise updates on a diverse group of topics in infectious diseases. Chapters cover newly recognized and emerging infectious diseases problems including Escherichia coli O157:H7, Helicobacter pylori, and Acinetobacter. Other chapters provide therapeutic updates of a range of problems including exacerbations of chronic obstructive pulmonary disease, HIV infection, and onychomycosis. Most reviews are relevant to the clinician with the exception of discussions on alternative treatments for methicillin-resistant Staphylococcus aureus (MRSA) and antibiotic-resistance genes in plants. There are 12 chapters, and most of the authors are recognized authorities in their areas. 

Page borders are color-coded by chapter with matching color schemes for tables. Each chapter contains a table of highlights with headings of “What’s in,” “What’s out,” and frequently “What’s controversial,” or “What’s needed.” This approach works with variable success. It does give a reader whose thumbing through the book a quick look at the major issues. But I can imagine authors struggling with what to include in this format leading to unhelpful entries such as “Over-prescribing of conventional antibiotics” under “What’s out” in a chapter on alternative treatments for MRSA. In rapidly changing areas including HIV therapeutics, what was “in” at the time of writing is already “out” or “controversial” by the time of this review in the fall of 2001. 

The chapters are, for the most part, well written and factual. The reviews on Clostridium difficile diarrhea (authored by the editor) and E. coli O157:H7 are especially well done. Unfortunately, the chapter on HIV chemotherapy contains a few inexplicable errors. Lopinavir, a protease inhibitor, is listed as a nucleoside reverse transcriptase inhibitor in both a table and the text. The authors also states that the nucleotide analogs, such as tenofovir are active in their native form. In fact, they are prodrugs that require phosphorylation by cellular enzymes. The HIV chapter is also the most dated, though I cannot fault the authors for this, given the dynamic nature of the field.

The editor does not tell us the intended audience for the book, but it appears to be geared for the infectious disease specialist rather than the generalist. The reviews average about six small pages of text, and the discussions are not sufficiently complete to serve as a background source for the uninitiated. The editor writes that review articles are often unwieldly or out-of-date at the time of publication. This text aims to summarize new information concisely. Concise it is – but perhaps too much so, as I came away from reading many of the reviews longing for more depth. Nonetheless, this volume generally succeeds with providing “fast facts” in a well-written and easy to read format.

